# Baseline Data on Viral Suppression, Treatment Adherence and Mental Health in Peripartum Girls and Young Women With HIV in Zimbabwe

**DOI:** 10.1002/jia2.70117

**Published:** 2026-06-19

**Authors:** Tariro D. Chawana‐Mutingwende, Bernard Ngara, Leon‐Say Mudadi, Thelma T. Tauya, Marvelous Sibanda, Mercy Mutambanengwe‐Jacob, Precious Andifasi, Bernadette Malunda, Tinashe Chidemo, Annie Mapfunde, Sandra Nyakudya, Anna Z. Mufumisi, Thandiwe H. Chirenda, Tobias Chitambo, Walter T. S. Nderecha, Constance Murandu, Miriam Gwande, Kudakwashe Muringayi, Rhoda Micah Bongo, Hideaki Okochi, Karen Kuncze, Angela Mushavi, Nicola Willis, Karen Webb, Monica Gandhi, Lynda Stranix‐Chibanda

**Affiliations:** ^1^ University of Zimbabwe Clinical Trials Research Centre Harare Zimbabwe; ^2^ Faculty of Medicine and Health Sciences University of Zimbabwe Harare Zimbabwe; ^3^ Ministry of Health and Child Care Harare Zimbabwe; ^4^ Division of HIV, Infectious Diseases and Global Medicine University of California San Francisco San Francisco California USA; ^5^ Zvandiri Harare Zimbabwe; ^6^ Organization for Public Health Interventions and Development (OPHID) Harare Zimbabwe

**Keywords:** adherence, adolescent girls and young women, HIV, peripartum, urine tenofovir levels, virological suppression

## Abstract

**Introduction:**

Peripartum adolescent girls and young women (AGYW) with HIV have lower viral suppression compared to older women. But the recently introduced dolutegravir‐based regimens and psychosocial peer motivation may improve viral control. We assessed viral suppression, adherence, drug exposure and mental health in a cohort of peripartum AGYW with HIV.

**Methods:**

We present baseline data from an ongoing randomized controlled and observational cohort study. Peripartum (conception to 24 months postpartum) AGYW 15‐ to 24‐year‐old with HIV and >3 months on tenofovir‐based antiretroviral therapy were enrolled between September 2024 and July 2025. Point‐of‐care viral load and urine tenofovir assays were performed at entry, as well as data on sociodemographic, HIV, treatment and mental health characteristics. Anxiety was assessed using the Generalized Anxiety Disorder‐7, depression using the Patient Health Questionnaire‐9, stigma using the Modified HIV Stigma Scale and post‐traumatic stress disorder using the Primary Care Post Traumatic Stress Disorder 5 tools.

**Results:**

We enrolled 151 participants. Mean age was 21.1 years. All participants were receiving tenofovir, lamivudine and dolutegravir. Baseline mean viral load was 1133 copies/mL (cpm); 135 (89.4%) were virally suppressed (viral load <40 cpm). Mean self‐reported adherence over the past 1 month was 97.5%. Tenofovir was detected in 141 (93.4%) participants at baseline. The following were significantly associated baseline with viral suppression (mean [SD]; 95% CI) or (*n* [%]): higher self‐reported adherence (99.5 [1.7]; 99.2−99.8, *p*<0.001), detectable urine tenofovir (133 [94.3]; *p*<0.001), being married (79 [92.9]; *p* = 0.03), having food security (86 [95.6]; *p* = 0.004) and being financially secure (74 [96.1]; *p* = 0.02). Seven (4.6%) experienced post‐traumatic stress disorder, 132 (88.0%) had minimal or no depression; 137 (92.0%) had minimal anxiety and 81 (53.6%) experienced moderate stigma. Participants without detectable tenofovir in urine were 0.05 times less likely to have baseline viral suppression, adjusted odds ratio (95% CI) (0.05 [0.006−0.4]; *p* = 0.004). Participants with higher self‐reported adherence were 1.3 times more likely to have viral suppression (1.3 [1.1−1.6]; *p* = 0.008).

**Conclusions:**

We found high viral suppression at enrolment into a cohort study in peripartum AGYW with HIV. Viral suppression was strongly associated with positive urine tenofovir assays and higher self‐reported adherence. High mental wellness was demonstrated.

## 1 Introduction

The scale‐up of universal antiretroviral therapy (ART) has significantly reduced HIV‐related morbidity and mortality. However, peripartum adolescent girls and young women (AGYW) with HIV lag behind; having lower viral suppression rates and less peripartum care engagement, ART access, adherence and retention compared to older women [1, 2, 3, 4, 5]. AGYW face challenges with long‐term ART adherence for sustained viral suppression, more so during the peripartum period. Moreover, about half of AGYW with HIV are diagnosed for the first time when presenting for their first antenatal care visit [6, 7], and a higher proportion of their infants acquire HIV compared to infants of older mothers (10.8 vs. 6.1%, respectively) [5, 8]. In Zimbabwe, 37%–55% of AGYW on ART exhibited viral loads above 1000 copies/mL (cpm), 67.6% of whom had clinically significant drug resistance to non‐nucleoside reverse transcriptase inhibitors (NNRTIs) before the rollout of dolutegravir due to poor adherence, a result of complex psychosocial factors unique to this group [9, 10, 11, 12, 13]. Interventions to improve treatment outcomes for adolescents and young women with HIV, and their infants, are crucial [11].

Literature reporting the effect of peer‐supported differentiated service delivery on treatment outcomes is conflicting. While some show substantial improvement in linkage to care [14, 15, 16, 17, 18], virological suppression [15, 19], retention in care and adherence [15, 18, 20, 21, 22], reducing rates of morbidity and mortality among peripartum AGYW with HIV [11, 19], some show mixed or no benefit at all [23]. In Zimbabwe, two peer support models are operational—the Zvandiri and Operation Triple Zero (OTZ) models [15, 17, 18]. AGYW who experienced the Zvandiri intervention showed improved virological suppression, retention in care and mental health wellness compared to those who did not [11, 17, 18]. The Adolescent Triple Zero (ATZ) intervention was adapted in Zimbabwe from the OTZ programme, and it engages peer supporters called Champions to empower young people to take charge of their own health and achieve zero missed appointments, zero missed pills and zero viraemia among adolescents and young people with HIV [15, 24]. Close conversations with peers and linkage to psychosocial support have the potential to make adolescents and young people open up more about their challenges [25].

Dolutegravir in combination with tenofovir disoproxil fumarate and lamivudine (TLD) has been the recommended first‐line ART regimen in Zimbabwe since 2019 [26, 27]. Dolutegravir, an integrase strand transfer inhibitor, has superior efficacy and tolerability compared to NNRTIs, with a high barrier to resistance [28, 29], which is important in populations with adherence challenges like AGYW. However, 6 years into the rollout of TLD, resistance to dolutegravir is emerging [30, 31, 32]. Fourteen percent of genotypes from people with HIV in Zimbabwe with viral loads ≥1000 cpm after at least 6 months on a dolutegravir‐based ART regimen showed high‐level dolutegravir resistance [33], with nucleotide reverse transcriptase inhibitors (NRTIs) and NNRTIs cross‐class resistance demonstrated. This is due to sub‐optimal adherence in pre‐existing NRTI and NNRTI resistance [34, 35]. Adequate adherence to ART remains key.

Measuring antiretroviral adherence accurately is challenging, and traditional methods are limited [36, 37]. Measuring antiretroviral concentrations in biomatrices such as plasma, red blood cells (dried blood spots—DBS), hair and more recently—urine—assesses adherence objectively and can be used to both determine drug exposure and support adherence [37]. Drug levels in plasma and urine represent adherence over the short‐term and can be impacted by white coat adherence dosing, while DBS and hair levels reflect cumulative ARV exposure over weeks to months [36, 37, 38, 39]. Although urine tenofovir levels reflect exposure over the past few days, the result is available in real‐time at the point‐of‐care (POC) with a recently developed lateral flow assay [40, 41, 42]. The result is available within 3‐5 min, enabling real‐time result feedback and potentially increasing motivation for adherence [43, 44, 45]. Additionally, urine samples are easy to collect compared to phlebotomy; urine drug levels provide information on both adherence and drug exposure in one assay; and the test is cheaper (<USD$2−3 per test kit) [46] than hair, plasma and DBS drug level tests [45, 47].

Viral load and drug level measurement POC technology create the opportunity to integrate POC monitoring into routine care to provide differentiated services to AGYW with viraemia [38, 48]. The World Health Organization (WHO) 2021 treatment monitoring guidelines advocate for POC viral load monitoring for pregnant women to expedite management of viraemia [49].

Within the context of recruitment for the ongoing “Promoting viral load suppression in peripartum adolescent girls and young women with HIV by integrating drug level‐informed enhanced adherence counselling into routine treatment monitoring (P‐PACT)” randomized clinical trial, we hypothesized that peripartum AGYW receiving current dolutegravir‐based ART regimens and receiving peer support in Zimbabwe would have high rates of viral suppression at the time of trial enrolment. Additionally, we hypothesized that detectable POC urine tenofovir levels at baseline would be associated with suppressed viral loads (below the level of detection of 40 cpm). Here, we present baseline sociodemographic, HIV and mental health characteristics of enrolled participants in P‐PACT, as well as the relationship between baseline urine tenofovir assay results and baseline viral load.

## 2 Methods

### 2.1 Study Population and Setting

We consecutively enrolled peripartum AGYW 15‐ to 24‐year‐old with HIV in Harare and Chitungwiza, two large urban areas in Zimbabwe. Participants were recruited from the national ART programme. All participants in this trial had to be on tenofovir‐based ART for at least 3 months. Participants were excluded if they were on anti‐tuberculosis (TB) treatment due to possible drug−drug interactions. The peripartum period was described as the period from conception up to 24 months post‐partum. All study procedures were conducted at Seke South Clinical Research Site (CRS) in Chitungwiza, one of the seven CRSs of the University of Zimbabwe Clinical Trials Research Centre (UZ‐CTRC). Screening for eligible participants was done between September 2024 and July 2025. Follow‐up of enrolled participants is ongoing. We present participants’ baseline data prior to randomization and study intervention.

### 2.2 Existing Support Programme for AGYW in Zimbabwe

All participants are supported by Zvandiri Community Adolescent Treatment Supporters (CATS) and Young Mentor Mums (YMMs). The Zvandiri group pioneered community‐based peer counselling and support by CATS and YMMs among 18‐ to 24‐year‐olds living with HIV to deliver adherence and psychosocial support during clinic reviews and through individualized community‐based support (text messages, phone calls and home visits), peer support groups and linkage to support services [11, 17, 18]. YMMs are trained young mothers who initially received training as CATS—they support other young mothers living with HIV, their infants and their partners during the pregnancy and breastfeeding period [50]. Frequency and type of contact and support is determined by whether participants are considered stable or in need of enhanced care. Support and contact are more intense for those with viral load >1000 cpm, are at risk of common mental disorders or major depressive disorders, missed clinic visits, had recently started ART, are pregnant or had other psychosocial challenges or protection challenges [11, 17, 50, 51]. For pregnant women and their families, services include mental health counselling, linkage to HIV testing and treatment, linkage to antenatal care and preparedness for delivery, access to viral load testing and early infant diagnosis, and adherence check‐in.

The Zvandiri YMM programme improved treatment outcomes, with 97.1% of the young mothers versus 66.2% in standard of care achieving viral suppression by the end of 2020; 96% of HIV‐exposed infants versus 64% in standard of care being tested for HIV and receiving their results; and perinatal HIV transmission among the infants was 1% versus 6.7% in standard of care [50, 52, 53, 54]. Other improvements were seen in mental health wellness, partner disclosure and testing, linkage to ART and pre‐exposure prophylaxis (PrEP), as well as relationships with partners [50].

### 2.3 POC Viral Load Testing

POC viral load testing was performed on the GeneXpert XVI module machine manufactured by Cepheid with a measuring range of 40−10,000,000 cpm. The machine can run 16 tests at a time [55]. Six millilitres (6 mL) of whole blood was collected into EDTA (purple top) tubes at Seke South CRS and transported to the UZ‐CTRC Central Laboratory, Harare, Zimbabwe, for testing (transit time 1 h). Results were sent online immediately after release from the UZ‐CTRC Central Laboratory to the site laboratory through the Laboratory Information System (LIS) system [56, 57], where they print automatically and go through the site's quality control checks before results are provided to the participants. Results were available to the participants on the same day before leaving the clinic. In this analysis, viral suppression was defined as viral load <40 cpm, being the lower limit of detection of the testing platform.

### 2.4 POC Tenofovir Testing in Urine

For POC urine tenofovir testing, 20−50 mL urine was self‐collected into sterile urine jars. Two to three drops were placed onto the test kit by trained study staff. One visible line on the urine tenofovir strip means tenofovir is detected, while two lines mean that tenofovir is not detected. The test is a rapid chromatographic immunoassay based on competitive binding [58]. Tenofovir levels below 1500 ng/mL will not saturate and read as not detected, while levels higher than 1500 ng/mL will be detected [58]. Compared to quantitative urine tenofovir concentrations, the POC tenofovir test is accurate at detecting tenofovir at the threshold of 1500 ng/mL, with a sensitivity of 96.1% and specificity of 95.2% [59]. We can assay tenofovir alone to judge adherence to the whole ART regimen because TLD is a fixed‐dose combination.

### 2.5 Data Collection Tools

Interviewer‐administered sociodemographic, medical history, self‐reported adherence, Modified HIV Stigma Scale [60, 61], Patient Health Questionnaire (PHQ‐9) [62], Generalized Anxiety Disorder (GAD‐7) [63] and Primary Care Post Traumatic Stress Disorder 5 (PC‐PTSD‐5) screening [64, 65] questionnaires were administered at baseline.

### 2.6 Study Outcomes

The primary outcome is baseline viral load suppression (viral load <40 cpm), and the secondary outcome is baseline detectable tenofovir in urine.

### 2.7 Ethical Considerations

Participants 18 years and older provided independent informed consent. Participants aged 15 years and younger than 18 years of age at screening/enrolment were considered emancipated minors based on pregnancy and giving birth; therefore, they provided independent informed consent. Parental consent was waived for this group.

This study was approved by the Joint Research Ethics Committee (JREC 328/2023), Medical Research Council of Zimbabwe (MRCZ/A/3089), Research Council of Zimbabwe (RCZ 04994), Harare City Council, Chitungwiza Council and Chitungwiza Hospital Institutional Review Boards. The study was registered with the Pan African Clinical Trials Registry (PACTR202508903618628).

### 2.8 Statistical Analysis

Data was entered into Research Electronic Data Capture (REDCap), a web‐based electronic database [66] and was analysed in Stata version 14 [67]. Baseline characteristics of participants were summarized using descriptive statistics. Continuous variables were presented as mean ± standard deviation (SD) or median (interquartile range [IQR]) where appropriate. Categorical variables were summarized as frequencies and percentages.

In the bivariate analysis, Pearson Chi‐square (and Fisher's test where appropriate) or Student's *t*‐test were used to determine association with viral load suppression. In multivariate analysis, we used the stepwise forward (*p* = 0.05) technique in performing regression. Logistic regression was used to determine variables associated with viral load suppression. Results were expressed as adjusted odds ratios (aOR) with 95% confidence intervals (CIs) and corresponding *p*‐values. Statistical significance was set at a 95% level (α = 0.05).

## 3 Results

### 3.1 Univariate Analysis

One hundred and fifty‐five participants were screened for eligibility, and 151 (97.4%) participants were recruited. Three (2.0%) were excluded because they were not on tenofovir, and one (0.6%) was excluded because she was on tenofovir for less than 3 months. None refused participation. Mean (SD) age was 21.1 (2.2) years (Table 1). Eighty‐five (56.3%) participants were married, and one (0.7%) participant was registered in school. Seventeen (11.3%) sometimes suffered from food insecurity in the previous 4 weeks, and 17 (11.3%) were fairly often financially/economically insecure.

**TABLE 1:** Baseline sociodemographic and HIV characteristics.

Variable	*N* (%) or mean (SD); range
	*n* = 151
**Sociodemographic characteristics**	
Age in years:	21.1 (2.2); 16−24
Age in years:	
15−19	35 (23.2)
20−24	116 (76.8)
Marital status:	
Married	85 (56.3)
Not married/single	23 (15.2)
Cohabiting	25 (16.6)
Divorced/separated	17 (11.3)
Widowed	1 (0.7)
Registered in school:	
Yes	1 (0.7)
No	150 (99.3)
Earning own income:	
Yes	52 (34.4)
No	99 (65.6)
Partner earning income:	
Yes	119 (78.8)
No	3 (2.0)
N/A	29 (19.2)
Religion:	
Christian	148 (98.0)
African Traditional Religion	0 (0.0)
Muslim	0 (0.0)
Not religious	3 (2.0)
Worried that she will not have enough food in the past 4 weeks:	
Never	90 (59.6)
Rarely (once or twice)	44 (29.1)
Sometimes (3–10 times)	17 (11.3)
Often (more than 10 times)	0 (0.0)
Did not have enough money in the household for rent, food or utilities, such as gas/electric:	
Never	77 (51.0)
Occasionally (1−2 times)	57 (37.8)
Fairly often (3−5 times)	17 (11.3)
Very often (six or more times)	0 (0.0)
Spent a night in jail/prison in entire lifetime:	
Yes	5 (3.3)
No	146 (96.7)
**HIV and treatment characteristics**	
Total time on ART in years (*n* = 133):	5.6 (5.6); 0.25−19.4
ART regimen:	
Tenofovir/lamivudine/dolutegravir	151 (100.0)
Time on tenofovir/lamivudine/dolutegravir in years (*n* = 123):	3.2 (3.0); 0.2−16
HIV viral load:	
Detectable	83 (54.3)
Undetectable	68 (45.7)
HIV viral load:	
<40 cpm (suppressed)	135 (89.4)
>40 cpm (unsuppressed)	16 (10.6)
HIV viral load (cpm):	1133 (7127.9); <40–72100
Self‐reported adherence in the past 1 month:	97.5 (11.6); 0−100
Urine tenofovir detectable:	
Yes	141 (93.4)
No	10 (6.6)
Mode of HIV acquisition:	
Perinatal	54 (35.8)
Sexual	96 (63.6)
Unknown	1 (0.7)
Disclosed HIV status to partner:	
Yes	112 (74.2)
No	10 (6.6)
N/A	29 (19.2)
Reasons for not disclosing to partner (*n* = 10):	
We did not know one another very well	1 (10.0)
Concerned this person would not understand	1 (10.0)
Did not know how to tell the person about my diagnosis	2 (20.0)
Worried the person would no longer like me after knowing	4 (40.0)
Concerned how this person would feel about me after knowing	2 (20.0)
Disclosed HIV status to someone other than partner:	
Yes	145 (96.0)
No	6 (4.0)
Reasons for not disclosing to someone other than partner:	
People may tell others	2 (33.3)
Did not know how to tell the person about my diagnosis	2 (33.3)
Have a right to privacy	1 (16.7)
Could not figure out how to talk about the diagnosis	1 (16.7)
Know partner's HIV status:	
Yes	110 (72.9)
No	12 (8.0)
N/A	29 (19.2)
Partner's HIV status (*n* = 110):	
Living with HIV	65 (59.1)
Not living with HIV	45 (40.9)
**Mental health characteristics**	
Primary care post‐traumatic stress disorder 5 (PC‐PTSD‐5) score:	
Yes (scores 1−5)	7 (4.6)
No (score 0)	144 (95.4)
Patient Health Questionnaire (PHQ)‐9 score (*n* = 150):	
0−4 (minimal or no depression)	132 (88.0)
5−9 (mild depression)	16 (10.6)
10−14 (moderate depression)	1 (0.7)
15−19 (moderately severe depression)	1 (0.7)
20−27 (severe depression)	0 (0.0)
Generalized Anxiety Disorder (GAD)‐7 (*n* = 149):	
0−4 (minimal anxiety)	137 (92.0)
5−9 (mild anxiety)	12 (8.0)
10−14 (moderate anxiety)	0 (0.0)
15−21 (severe anxiety)	0 (0.0)
Modified HIV stigma scale score:	
Low‐level stigma (10−20)	70 (46.4)
Middle‐level stigma (21−30)	81 (53.6)
High‐level stigma (31−40)	0 (0.0)

*Notes*: Primary care post‐traumatic stress disorder 5 (PC‐PTSD‐5) tool was used, scored from 0 to 5. Stigma was measured using the modified HIV stigma scale and was scored from 0 to 40.

Abbreviations: ART, antiretroviral therapy; cpm, copies/mL; GAD‐7, Generalized Anxiety Disorder 7; PC‐PTSD‐5, primary care post‐traumatic stress disorder 5; PHQ‐9, Patient Health Questionnaire 9.

^a^ Viral load lower limit of detection is 40 copies/mL.

^b^ Self‐reported adherence was measured by the Visual Analogue Scale (VAS) on a scale of 0−100.

^c^ Mode of HIV acquisition was self‐reported by participants.

All participants were on TLD, and the mean (SD) time on this regimen was 3.2 (3.0) years. Mean (SD) self‐reported adherence over the past 1 month was 97.5% (11.6). Ninety‐six (63.6%) participants self‐reported having acquired HIV through sexual intercourse; 112 (74.2%) participants disclosed their HIV status to their partners, and 65 (59.1%) partners were living with HIV.

Viral load was detectable in 83 (54.3%) participants at baseline. Mean viral load was 1133 cpm; 135 (89.4%) were virally suppressed (viral load <40 cpm). Tenofovir was detected at baseline in 141 (93.4%) participants via the urine tenofovir assay. At baseline, seven (4.6%) reported experiencing post‐traumatic stress disorder, 132 (88.0%) had minimal or no depression; 137 (92.0%) had minimal anxiety and 81 (53.6%) experienced moderate stigma.

### 3.2 Bivariate Analysis

The following variables were significantly associated with viral suppression at baseline (Table 2): being married *n* (%) (79 [92.9]; *p* = 0.03); being food secure *n* (%) 86 (95.6); *p* = 0.004; being financially/economically secure *n* (%) 74 (96.1); *p* = 0.02; self‐reported high adherence over the past 1 month mean (SD); 95% CI (99.5 [1.7]; 99.2−99.8, *p*<0.001) and detectable tenofovir in urine, *n* (%) (133 [94.3]; *p*<0.001). Post‐traumatic stress disorder, depression, anxiety and stigma were not associated with viral suppression.

**TABLE 2:** Comparison of participant characteristics by baseline viral load.

Variable	Viral load <40 cpm	Viral load >40 cpm	*p*‐value
	*N* (%) or mean (SD);	*N* (%) or mean (SD);	
	95% CI	95% CI	
	*n* = 135	*n* = 16	
**Sociodemographic characteristics**			
Age in years:	21.1 (2.2): 20.8−21.5	21.1 (2.1): 19.9−22.2	0.4
Age in years:			
15−19	30 (85.7)	5 (14.3)	0.4
20−24	105 (90.5)	11 (9.5)	
Marital status:			
Married	79 (92.9)	6 (7.1)	0.03
Not married/single	20 (87.0)	3 (13.0)	
Cohabiting	21 (84.0)	4 (16.0)	
Divorced/separated	15 (88.2)	2 (11.8)	
Widowed	0 (0)	1 (100.0)	
Registered in school:			
Yes	0 (0.0)	1 (100.0)	0.004
No	135 (90.0)	15 (10.0)	
Earning own income:			
Yes	47 (90.4)	5 (9.6)	0.8
No	88 (88.9)	11 (11.1)	
Partner earning income:			
Yes	108 (90.8)	11 (9.8)	0.4
No	2 (66.7)	1 (33.3)	
N/A	25 (86.2)	4 (13.8)	
Religion:			
Christian	132 (89.2)	16 (10.8)	0.5
African Traditional Religion	0 (0.0)	0 (0.0)	
Muslim	0 (0.0)	0 (0.0)	
Not religious	3 (100.0)	0 (0.0)	
Worried that she will not have enough food in the past 4 weeks:			
Never	86 (95.6)	4 (4.4)	0.004
Rarely (once or twice)	37 (84.1)	7 (15.9)	
Sometimes (3–10 times)	12 (70.6)	5 (29.4)	
Often (more than 10 times)	0 (0.0)	0 (0.0)	
Did not have enough money in the household for rent, food or utilities, such as gas/electric:			
Never	74 (96.1)	3 (3.9)	0.02
Occasionally (1−2 times)	48 (84.2)	9 (15.8)	
Fairly often (3−5 times)	13 (76.5)	4 (23.5)	
Very often (six or more times)	0 (0.0)	0 (0.0)	
Spent a night in jail/prison in entire lifetime:			
Yes	2 (40.0)	3 (60.0)	0.8
No	66 (45.2)	80 (54.8)	
**HIV and treatment characteristics**			
Total time on ART in years:	5.7 (5.6); 4.6−6.6	4.8 (6.0); 1.1−8.6	0.3
ART regimen:			
Tenofovir/lamivudine/dolutegravir	68 (45.0)	83 (55.0)	—
Time on tenofovir/lamivudine/dolutegravir in years:	3.2 (2.9); 2.7−3.8	2.7 (3.2); 0.5−4.8	0.3
Self‐reported adherence in the past 1 month:	99.5 (1.7); 99.2−99.8	81.0 (31.5); 64.2−97.8	<0.001
Urine tenofovir detectable:			
Yes	133 (94.3)	8 (5.7)	<0.001
No	2 (20.0)	8 (80.0)	
Mode of HIV acquisition:			
Perinatal	47 (87.0)	7 (13.0)	0.8
Sexual	87 (90.6)	9 (9.4)	
Unknown	1 (100.0)	0 (0.0)	
Disclosed HIV status to partner:			
Yes	102 (91.1)	10 (8.9)	0.5
No	8 (80.0)	2 (20.0)	
N/A	25 (86.2)	4 (13.8)	
Disclosed HIV status to someone other than partner:			
Yes	130 (89.7)	15 (10.3)	0.6
No	5 (83.3)	1 (16.7)	
Know partner's HIV status:			
Yes	100 (90.9)	10 (9.1)	0.6
No	10 (83.3)	2 (16.7)	
N/A	25 (86.2)	4 (13.8)	
Partner's HIV status:			
Living with HIV	60 (92.3)	5 (7.7)	0.5
Not living with HIV	40 (88.9)	5 (11.1)	
**Mental health characteristics**			
Primary care post‐traumatic stress disorder 5 (PC‐PTSD‐5) score:			
Yes (scores 1−5)	5 (71.4)	2 (28.6)	0.1
No (score 0)	14 (9.7)	130 (90.3)	
Patient Health Questionnaire			
(PHQ)‐9 score (*n* = 150):	119 (90.2)	13 (9.9)	0.7
0−4 (minimal or no depression)	13 (81.3)	3 (18.7)	
5−9 (mild depression)	1 (100.0)	0 (0.0)	
10−14 (moderate depression)	1 (100.0)	0 (0.0)	
15−19 (moderately severe depression)	0 (0.0)	0 (0.0)	
20−27 (severe depression)			
(GAD)‐7 (*n* = 149):	124 (90.5)	13 (9.5)	0.1
0−4 (minimal anxiety)	9 (75.0)	3 (25.0)	
5−9 (mild anxiety)	0 (0.0)	0 (0.0)	
10−14 (moderate anxiety)	0 (0.0)	0 (0.0)	
15−21 (severe anxiety)			
Modified HIV stigma scale score:			
Low‐level stigma (10−20)	66 (94.3)	4 (5.7)	0.07
Middle‐level stigma (21−30)	69 (85.2)	12 (14.8)	
High‐level stigma (31−40)	0 (0.0)	0(0.0)	

*Notes*: Primary care post‐traumatic stress disorder 5 (PC‐PTSD‐5) tool was used, scored from 0 to 5. Stigma was measured using the modified HIV stigma scale and was scored from 0 to 40.

Abbreviations: ART, antiretroviral therapy; cpm, copies/mL; GAD‐7, Generalized Anxiety Disorder 7; PC‐PTSD‐5, primary care post‐traumatic stress disorder 5; PHQ‐9, Patient Health Questionnaire 9.

^a^Viral load lower limit of detection is 40 copies/mL.

^b^Self‐reported adherence was measured by the Visual Analogue Scale (VAS) on a scale of 0−100.

^c^Mode of HIV acquisition was self‐reported by participants.

### 3.3 Multivariate Analysis

In multivariate analysis, participants with undetectable tenofovir in urine were 0.05 times less likely to have viral suppression at baseline, aOR (95% CI) (0.05 [0.006−0.4]; *p* = 0.004). Participants with high self‐reported adherence were 1.3 times more likely to have viral suppression, aOR (95% CI) (1.3 [1.1−1.6]; *p* = 0.008).

## 4 Discussion

Our study found high viral suppression at baseline and a strong association with urine tenofovir levels with suppression. Eighty‐nine percent of the cohort had viral suppression using a strict cut‐off of <40 cpm. This finding differs from previous studies, which found high viraemia and virological treatment failure among AGYW [4, 10]. In 2019, 65% adolescents 10−19 years old in Zimbabwe had viral suppression (viral load <1000 cpm) on NNRTI‐based ART [68], while 47% adolescents 13−19 years old had viral suppression on the same regimen and using the same viral load cut‐off [11]. On the other hand, 87% AGYW 15−24 years old on NNRTI‐based ART were virally suppressed (viral load <1000 cpm) at the beginning of the YMM programme in 2019 [69], which is comparable to our findings.

Causes of high viral suppression in our cohort could be attributed to several factors. First, dolutegravir was rolled out in Zimbabwe in September 2019 in adults and in February 2022 in children [26, 27], and all our participants were taking it. Dolutegravir is known to sustain viral suppression even at adherence rates lower than 100% (i.e. it is more forgiving) and has a high genetic barrier against resistance [28, 29, 70]. Two studies in rural Zimbabwe found viral suppression (viral load <1000 cpm) increased from 76%–80% to 95%–96% among children and adolescents 6 months after switching from NNRTI‐based ART to dolutegravir‐based ART [71, 72], but the sustainability of this degree of viral suppression is uncertain. A study in South Africa found that at 6 months, dolutegravir‐based ART had higher viral suppression (viral load <50 cpm) (66%) compared to efavirenz‐based ART (54%); but at 12 months, viral suppression was comparable between the two groups (70% vs. 68%, respectively). Additionally, the high adherence and drug exposure that are demonstrated in our study make it impossible to distinguish between viral suppression from dolutegravir's forgiving nature and that of high adherence.

Second, we recruited participants who were in the Zvandiri programme. All AGYW in the programme are closely supported by CATS to adhere to treatment and care. During the peripartum period, they are supported by YMMs. Previous studies have shown improved viral suppression as well as improved linkage to services and retention in care, improved adherence and improved psychosocial wellbeing using the Zvandiri model [69, 73]. The Zvandiri trial found that young people in the programme were 42% more likely to have suppressed viral loads (viral load <1000 cpm) compared to standard of care [11]. The Zvandiri model is likely to be increasing viral suppression in our cohort.

We found that food and financial/economic security were associated with viral suppression in our study. This is consistent with previous studies that found that social protection improved viral suppression [13, 74, 75, 76, 77]. In this study, two‐thirds of the women were earning their own income, as well as 80% of their partners. In the Zvandiri programme, social protection comes in the form of economic strengthening where AGYW are empowered with vocational training, career guidance and work‐readiness training to generate income in the Skills2Live project [78]. Prior to the intervention, three‐quarters of AGYW in a similar setting were unemployed and had poor virological control [69]. Social protection might have contributed to high viral load suppression in this study.

Third, the Zimbabwe national ART programme is supported by the Organization for Public Health Interventions and Development (OPHID), a local non‐profit organization that implements approaches and strategies to improve HIV services. OPHID develops innovative solutions to health, including retention in care [79]. Our participants were all under OPHID care, and this could be why we have high viral suppression in our cohort.

High rates of detectable tenofovir in urine and high self‐reported adherence were strongly associated with viral suppression in our study. To our knowledge, our study is the first to evaluate the urine tenofovir POC test in peripartum AGYW with HIV and link this test to virological outcomes in this group. Urine tenofovir measurement provides an objective measure of adherence to ART. Detecting lower drug levels in urine and viraemia creates openings for conversations about adherence challenges, while normal drug levels in the presence of viraemia, however, could signify white coat effect or drug resistance [58].

We also found encouraging mental health data at baseline in our cohort. Almost all the participants reported no, minimal or mild post‐traumatic stress disorder, depression and anxiety. Although this data conflicts with previous literature, which showed poor mental health in AGYW [80], it, however, correlates with mental health outcomes after the Zvandiri model. The study found high rates of poor mental health in young mothers at baseline when enrolled in the Zvandiri trial, but this improved significantly when connected with a CATS or YMM [69]. A second trial found a 60% reduction in symptoms of common mental disorder after the Zvandiri intervention [73]. Close peer support may improve the mental health of peripartum AGYW. Mental health disorders were associated with decreased adherence in a previous study [18]. High mental wellness reported in this study could have increased adherence to ART, tenofovir exposure and viral suppression. On the other hand, about half of the women in this study experienced middle‐level stigma, which is high. This reflects the need to include families and communities in the fight against HIV.

About two‐thirds had acquired HIV through sexual intercourse, while two‐thirds of the participants’ partners were living with HIV, highlighting the need for continued sexual reproductive health in this group to prevent new HIV acquisition. Three‐quarters of the participants had disclosed their HIV status to their partners, while almost all the women had disclosed to someone. This finding is encouraging. Previous studies showed high proportions of non‐disclosure in a similar setting, for fear of rejection [11, 69, 81, 82]. These findings were prior to the implementation of the Zvandiri intervention. High rates of disclosure in this study could be a result of the Zvandiri intervention, which is known to improve psychosocial wellbeing (confidence, self‐esteem and self‐worth) [18], which might lead to improved disclosure. This, however, needs to be evaluated further. Only one participant was registered in school in this group. This could be a result of the older nature of the group (mean age 21 years old). The participants might have completed their education training prior to enrolment into this study.

Our study has some strengths. It demonstrates the feasibility of POC technologies in monitoring viral outcomes in special populations in resource‐limited settings. Viral load results were available to the participants before leaving the clinic, and urine tenofovir results were available in real time. Decisions on the participants’ care can, therefore, be made timeously. Additionally, we assessed viral suppression in peripartum AGYW, a group that is at risk of non‐suppression. Findings from our study can inform care of peripartum AGYW in other settings to improve viral suppression and reduce perinatal HIV acquisition in infants. We also used a tight cut‐off of viral suppression of 40 cpm. High viral suppression using this tight cut‐off in peripartum AGYW is very encouraging. This study also has some weaknesses. We only presented baseline data from a clinical trial where participants are being followed for 6 months. We cannot ascertain if urine tenofovir levels can predict viral load outcomes, long‐term exposure to tenofovir and drug resistance. Additionally, we cannot ascertain the sustainability of viral suppression after the recent US funding cuts to HIV programmes. There is a need for longitudinal studies of dolutegravir and peer support, so that we can explore what role peer support plays (or not) as dolutegravir adherence fluctuates in young people.

## 5 Conclusions

We found high viral suppression in peripartum AGYW with HIV. Viral suppression was strongly associated with detectable tenofovir levels and high self‐reported adherence. We also found high mental wellness. Longitudinal studies and ascertaining which factors contributed to viral suppression and mental health in this important group are needed.

## Author Contributions

Conceptualization – TDC‐M, BN, L‐SM, MMJ, PA, NW, KW and LS‐C; Methodology – TDC‐M, BN, L‐SM, TTT, MS, MMJ, PA, BM, TC, RMB, HO, KK, NW, KW, MG and LS‐C; Software – TDC‐M and LS‐C; Data curation – TDC‐M, BN, L‐SM, TTT, MS, MMJ, PA, BM, TC and LS‐C; Investigation – TDC‐M, BN, L‐SM, TTT, MS, MMJ, PA, BM, TC; AM, SN, AZM, THC, TC, WTSN, CM, MG, KM, RMB, HO, KK, AM, NW, KW, MG and LS‐C; Validation – TDC‐M and LS‐C; Formal analysis – TDC‐M, BN, L‐SM, TTT, MS, MMJ, BM, TC; AM, SN, AZM, THC, TC, WTSN, CM, MG, KM, RMB, HO, KK, AM, NW, KW, MG and LS‐C; Supervision – TDC‐M and LS‐C; Funding acquisition – TDC‐M, RMB, HO, KK, MG and LS‐C; Visualization – TDC‐M and LS‐C; Project management – TDC‐M and LS‐C; Resources – TDC‐M and LS‐C; Writing – original draft – TDC‐M, BN, L‐SM, TTT, MS, MMJ, PA, BM, TC; AM, SN, AZM, THC, TC, WTSN, CM, MG, KM, RMB, HO, KK, AM, NW, KW, MG and LS‐C; Writing – reviewing and editing – TDC‐M, BN, L‐SM, TTT, MS, MMJ, PA, BM, TC; AM, SN, AZM, THC, TC, WTSN, CM, MG, KM, RMB, HO, KK, AM, NW, KW, MG and LS‐C. All authors have read and approved the final manuscript.

## Conflicts of Interest

All authors declare no conflicts of interest.

## Data Availability

The data that support the findings of this study are available on request from the corresponding author. The data are not publicly available due to privacy or ethical restrictions.
